# Uptake of orphan drugs in the WHO essential medicines lists

**DOI:** 10.2471/BLT.23.289731

**Published:** 2023-10-31

**Authors:** Enrico Costa, Lorenzo Moja, Veronika J Wirtz, Hendrika A van den Ham, Benedikt Huttner, Nicola Magrini, Hubert GM Leufkens

**Affiliations:** a WHO Collaborating Centre for Pharmaceutical Policy and Regulation, Department of Pharmacoepidemiology, Utrecht University, Utrecht, Kingdom of the Netherlands.; b Secretariat of the Expert Committee on the Selection and Use of Essential Medicines, Department of Health Products Policy and Standards, World Health Organization, 20 Avenue Appia, 1211 Geneva, Switzerland.; c WHO Collaborating Centre in Pharmaceutical Policy, Department of Global Health, Boston University, Boston, United States of America.; d WHO Collaborating Centre for Evidence-Based Research Synthesis and Guideline Development, Emilia-Romagna Health Authority, Bologna, Italy.

## Abstract

**Objective:**

We evaluated the uptake of medicines licensed as orphan drugs by the United States Food and Drug Administration (FDA) or European Medicines Agency (EMA) into the *WHO Model list of essential medicines* and the *WHO Model list of essential medicines for children* from 1977 to 2021.

**Methods:**

We collated and analysed data on drug characteristics, reasons for adding or rejecting medicines, and time between regulatory approval and inclusion in the lists. We compared trends in listing orphan drugs before and after revisions to the inclusion criteria of the essential medicines lists in 2001, as well as differences in trends for listing orphan and non-orphan drugs, respectively.

**Findings:**

The proportion of orphan drugs in the essential medicines lists increased from 1.9% (4/208) in 1977 to 14.6% (70/478) in 2021. While orphan drugs for communicable diseases have remained stable over time, we observed a considerable shift towards more orphan drugs for noncommunicable diseases, particularly for cancer. The median period for inclusion in the essential medicines lists after either FDA or EMA first approval was 13.5 years (range: 1–28 years). Limited clinical evidence base and uncertainty about the magnitude of net benefit were the most frequent reasons to reject proposals to add new orphan drugs to the essential medicines lists.

**Conclusion:**

Despite lack of a global definition of rare diseases, the essential medicines lists have broadened their scope to include medicines for rare conditions. However, the high costs of many listed orphan drugs pose accessibility and reimbursement challenges in resource-constrained settings.

## Résumé

**Objectif:**

Évaluer l'intégration des médicaments orphelins autorisés par la Food and Drug Administration (FDA) des États-Unis ou par l'Agence européenne des Médicaments (EMA) dans la *Liste modèle des médicaments essentiels* et la *Liste modèle des médicaments essentiels destinés à l'enfant*, toutes deux établies par l'OMS, entre 1977 et 2021.

**Méthodes:**

Nous avons collecté et analysé les données sur les caractéristiques des médicaments, les raisons pour lesquelles ils étaient acceptés ou refusés, et le délai entre leur autorisation réglementaire et leur ajout dans les listes. Nous avons comparé les tendances dans la mention des médicaments orphelins avant et après révision des critères d'inclusion dans les listes de médicaments essentiels en 2001, ainsi que les différences d'intégration dans les listes des médicaments orphelins d'une part et non orphelins de l'autre.

**Résultats:**

Le pourcentage de médicaments orphelins dans les listes de médicaments essentiels a augmenté, passant de 1,9% (4/208) en 1977 à 14,6% (70/478) en 2021. Alors que les médicaments orphelins pour les maladies transmissibles sont restés stables au fil du temps, nous avons observé une évolution considérable des médicaments orphelins pour les maladies non transmissibles, en particulier le cancer. Le délai médian d'inclusion au sein de la liste de médicaments essentiels après l'autorisation initiale de la FDA ou de l'EMA était de 13,5 ans (écart: 1 à 28 ans). Les données cliniques limitées et l'incertitude entourant l'ampleur des avantages réels figuraient parmi les raisons les plus fréquentes de refus des propositions d'ajout de nouveaux médicaments orphelins aux listes de médicaments essentiels.

**Conclusion:**

Malgré l’absence de définition globale des maladies rares, les listes de médicaments essentiels ont élargi leur champ d'application afin d'inclure des substances destinées au traitement d'affections rares. Cependant, les coûts élevés de la plupart des médicaments orphelins repris dans ces listes posent des problèmes d'accessibilité et de remboursement dans les milieux où les ressources sont limitées.

## Resumen

**Objetivo:**

Se evaluó la incorporación de los medicamentos autorizados como huérfanos por la Administración de Alimentos y Medicamentos (FDA) de los Estados Unidos o la Agencia Europea de Medicamentos (EMA) a la *Lista modelo de medicamentos esenciales de la OMS* y a la *Lista modelo de medicamentos esenciales para niños de la OMS* desde 1977 hasta 2021.

**Métodos:**

Se cotejaron y analizaron datos sobre las características de los medicamentos, las razones para añadir o rechazar medicamentos y el tiempo transcurrido entre la aprobación reglamentaria y la inclusión en las listas. Se compararon las tendencias de inclusión de medicamentos huérfanos antes y después de la revisión de los criterios de inclusión en las listas de medicamentos esenciales en 2001, así como las diferencias en las tendencias de inclusión de medicamentos huérfanos y no huérfanos, respectivamente.

**Resultados:**

El porcentaje de medicamentos huérfanos en las listas de medicamentos esenciales aumentó del 1,9% (4/208) en 1977 al 14,6% (70/478) en 2021. Mientras que los medicamentos huérfanos para enfermedades transmisibles han permanecido estables a lo largo del tiempo, se observa un cambio considerable hacia más medicamentos huérfanos para enfermedades no transmisibles, en particular para el cáncer. La mediana del periodo de inclusión en las listas de medicamentos esenciales tras la primera aprobación de la FDA o la EMA fue de 13,5 años (rango: 1-28 años). Una base de evidencias clínicas limitada y la incertidumbre sobre la magnitud del beneficio neto fueron las razones más frecuentes para rechazar las propuestas de añadir nuevos medicamentos huérfanos a las listas de medicamentos esenciales.

**Conclusión:**

A pesar de la falta de una definición global de enfermedades poco frecuentes, las listas de medicamentos esenciales han ampliado su ámbito de aplicación para incluir medicamentos para afecciones poco frecuentes. No obstante, el elevado coste de muchos medicamentos huérfanos incluidos en las listas plantea problemas de accesibilidad y reembolso en entornos con recursos limitados.

## ملخص

الغرض قمنا بتقييم إمكانية إضافة الأدوية المرخصة كأدوية مهملة بواسطة إدارة الغذاء والدواء الأمريكية (FDA)، أو وكالة الأدوية الأوروبية (EMA)، في القائمة النموذجية لمنظمة الصحة العالمية للأدوية الأساسية والقائمة النموذجية لمنظمة الصحة العالمية للأدوية الأساسية للأطفال من عام 1977 إلى عام 2021.

الطريقة قمنا بجمع وتحليل البيانات المتعلقة بخصائص الدواء، وأسباب إضافة أو رفض الأدوية، والمدة بين الموافقة التنظيمية والإدراج في القوائم. وقمنا بالمقارنة بين اتجاهات إدراج الأدوية اليتيمة قبل وبعد المراجعات، ومعايير الإدراج في قوائم الأدوية الأساسية في عام 2001، فضلًا عن الاختلافات في اتجاهات إدراج الأدوية المهملة وغير المهملة، على الترتيب.

النتائج ارتفعت نسبة الأدوية المهملة في قوائم الأدوية الأساسية من %1.9 (4/208) في عام 1977، إلى %14.6 (70/478) في عام 2021. وبينما ظلت الأدوية المهملة الخاصة بالأمراض المعدية مستقرة مع مرور الوقت، لاحظنا تحولًا كبيرًا نحو المزيد من الأدوية المهملة للأمراض غير المعدية، وخاصة السرطان. كانت الفترة المتوسطة للإدراج في قوائم الأدوية الأساسية بعد الموافقة الأولى من إدارة الغذاء والدواء (FDA)، أو وكالة الأدوية الأوروبية (EMA) هي 13.5 سنة (المدى: 1 إلى 28 عامًا). وكانت قاعدة الأدلة الإكلينيكية المحدودة وعدم اليقين بشأن حجم الفائدة الصافية، هما الأسباب الأكثر شيوعاً لرفض اقتراحات إضافة أدوية مهملة جديدة إلى قوائم الأدوية الأساسية.

الاستنتاج على الرغم من الافتقار لتعريف عالمي للأمراض النادرة، إلا أن قوائم الأدوية الأساسية قد قامت بتوسيع نطاقها لتشمل أدوية الحالات النادرة. ومع ذلك، فإن التكاليف المرتفعة للعديد من الأدوية المهملة المدرجة تشكل تحديات في إمكانية الحصول عليها والسداد في البيئات محدودة الموارد.

## 摘要

**目的:**

我们评估了 1977 年至 2021 年期间经美国食品和药物管理局 (FDA) 或欧洲药品管理局 (EMA) 批准的罕见病药物被纳入*世界卫生组织 (WHO) 基本药物标准清单*和*世界卫生组织 (WHO) 儿童基本药物标准清单*的情况。

**方法:**

我们整理并分析了有关药物特性的数据、增加或拒绝药物纳入的原因以及经监管部门批准后到纳入药物清单之间的时间间隔。我们比较了在 2001 年修订基本药物清单纳入标准前后罕见病药物的纳入趋势，并分别列举了罕见病药物和非罕见病药物纳入趋势的差异。

**结果:**

罕见病药物在基本药物清单中的比例由 1977 年的 1.9% (4/208) 增加到 2021 年的 14.6% (70/478)。虽然随着时间的推移，针对传染病的罕见病药物纳入情况一直保持稳定，但我们观察到，越来越多针对非传染性疾病（特别是癌症）的罕见病药物纳入情况发生了相当大的变化。经 FDA 或 EMA 首次批准后纳入基本药物清单的平均时间间隔为 13.5 年（范围：1-28 年）。有限的临床证据基础和净收益水平的不确定性是拒绝将新的罕见病药物纳入基本药物清单的最常见原因。

**结论:**

尽管罕见病缺乏全球性的定义，但基本药物清单已经扩大了其范围，将治疗罕见病的药物包括在内。然而，在资源受限环境中，许多纳入的罕见病药物成本高昂，在可及性和报销方面面临很大的挑战。

## Резюме

**Цель:**

Проведена оценка включения лекарственных средств, получивших лицензию Управления по контролю за пищевыми продуктами и лекарствами Соединенных Штатов Америки (FDA) или Европейского агентства по лекарственным средствам (EMA) как лекарственные средства для лечения редких болезней, в *Примерный перечень ВОЗ основных лекарственных средств* и *Примерный перечень ВОЗ основных лекарственных средств для детей* с 1977 по 2021 год.

**Методы:**

Были собраны и проанализированы данные о характеристиках препаратов, причинах их включения или невключения в перечни, а также о времени, прошедшем с момента одобрения препарата регуляторным органом до включения его в перечни. Было проведено сравнение тенденций включения лекарственных средств для лечения редких болезней до и после пересмотра критериев включения в перечни основных лекарственных средств в 2001 г., а также различий в тенденциях включения лекарственных средств для лечения редких болезней и лекарственных средств, не являющихся таковыми, соответственно.

**Результаты:**

Доля лекарственных средств для лечения редких болезней в перечнях ЖНВЛП увеличилась с 1,9% (4/208) в 1977 году до 14,6% (70/478) в 2021 году. В то время как количество лекарственных средств для лечения редких болезней остается неизменным, наблюдается значительный сдвиг в сторону увеличения количества лекарственных средств для лечения неинфекционных заболеваний, особенно онкологических. Медиана периода включения в списки основных лекарственных средств после первого одобрения FDA или EMA составила 13,5 года (диапазон: 1–28 лет). К наиболее частым причинам отклонения предложений о включении новых лекарственных средств для лечения редких болезней в перечни основных лекарственных средств относятся ограниченность клинической доказательной базы и неопределенность в отношении величины чистой пользы.

**Вывод:**

Несмотря на отсутствие международного определения редких болезней, перечни основных лекарственных средств расширены за счет включения в них препаратов для лечения редких болезней. Однако высокая стоимость большинства включенных в перечень лекарственных средств для лечения редких болезней приводит к возникновению сложностей с их доступностью и возмещением расходов в условиях ограниченных ресурсов.

## Introduction

For decades, pharmaceutical developers did not prioritize or see treatments for rare diseases as lucrative investments. Shifts in investment occurred in 1983 when the Orphan Drug Act was passed in the United States of America (USA). This act served as a portfolio of regulatory and financial incentives and tools to enhance investment into the development of medicines for rare diseases.^1^ Similar incentivizing legislation was adopted in Singapore in 1991, Japan in 1993, Australia in 1994 and in the European Union (EU) in 2000.^2^ However, such policy has also been criticized for creating large incentives that could promote interventions for rare diseases when there is limited or conflicting evidence.^3^

Over the past two decades, there has been a significant rise in the approval of medications for rare diseases, commonly known as orphan drugs, by both the United States Food and Drug Administration (FDA) and the European Medicines Agency (EMA).^4^^,^^5^ These drugs have provided notable clinical benefits to patients with rare diseases that previously had no treatment options. However, they have also sparked concerns due to their high costs and the limited evidence available when they are granted marketing authorization.^6^^,^^7^ The progress in developing new therapies for rare diseases has primarily benefited high-income countries, but these therapies may also help patients in low- and middle-income countries, where the adoption of these medications is gradually increasing.^8^^,^^9^

In a world marked by disparities in health-care access, the *WHO Model list of essential medicines* actively promotes access to essential medications globally, thereby contributing to universal health coverage (UHC).^10^ The list was created as a tool to help countries promote appropriate selection and use of effective and safe medicines in accordance with local health priorities and contingencies, to further the development of their own national essential medicines lists.^11^ The list prioritizes medicines delivering a high level of benefit to patients while rejecting those with uncertain benefits.^12^^,^^13^

In 2001, the World Health Organization (WHO) removed the expression “the majority of the population”, with the consent of the Member States, from the definition of essential medicines, re-defined as those that “satisfy the priority health-care needs of the population; selected with due regard to disease prevalence and public health relevance; evidence on efficacy and safety; and comparative cost-effectiveness.”^14^ Affordability changed from a precondition into a consequence of the drug procurement process, meaning that price alone was no longer considered a benchmark to accept or reject a medicinal product for inclusion into the essential medicines list.^15^ Despite different conceptual and legal frameworks (Table 1), orphan legislations and the essential medicines list can both be considered as guidelines on how to prioritize resources and allocate incentives towards universal public health needs; such as programmes to address treatment gaps in regions with few resources.^17^^,^^18^

**Table 1:** Regulatory frameworks of orphan drugs and essential medicines

Type	United States orphan drugs	EU orphan medicinal products	WHO essential medicines
Reference	Orphan Drug Act 1983^1^	Regulation (EC) 141/2000^4^	*WHO Model list of essential medicines*; TRS; No. 615 (1977)^16^ Revision of criteria: WHO medicines strategy EB109/8 resolution 2001
Definition	Drug treatments for conditions affecting less than 200 000 people or those drugs that will not be profitable within 7 years following approval	Medicines for the treatment, prevention or diagnosis of life-threatening or chronically debilitating diseases affecting < 5 in 10 000 people; for which no satisfactory treatments are authorized; if a treatment exists the new drug must be of significant benefit to those chronically infected	Drugs that satisfy the priority health-care needs of the population; increased magnitude of benefit
Perspective	From individual health to public health	From individual health to public health	From public health to individual health
Target	High-income countries	High-income countries	Mostly middle- and low-income countries
Purpose	New therapeutic options to treat rare diseases	New therapeutic options to treat rare diseases	Provide effective, safe and affordable medicines to as many people as possible
Incentives	7-year market exclusivity; 50% tax credit on clinical trials; technical assistance and accelerated evaluation; grant funding	10-year market exclusivity and fee reductions; technical assistance and accelerated evaluation	Tax reductions and/or exemptions at national level; increasing likelihood of reimbursement by public payers; possibility of waivers or donations (e.g. malaria)
Selection	Disease-driven	Disease-driven; demonstrated benefit beyond existing therapies is required	Drug-driven; closer integration with WHO guidelines increasingly pursued for antibiotics and oncological drugs
Clinical evidence	Pivotal clinical trials, controlled and uncontrolled cohort studies, case series	Pivotal clinical trials, controlled and uncontrolled cohort studies, case series	Systematic reviews and meta-analyses of clinical trials; evidence from field testing treatments

EU: European Union; RCT: randomized controlled trials; WHO: World Health Organization.

Here we quantify the type of medicines licensed as orphan drugs by either the FDA or EMA in the essential medicines lists in the period 1977–2021, comparing these trends before and after the 2001 WHO revision of the criteria for inclusion of essential medicines in the lists.

## Methods

### Data collection

We collated information on essential medicines from both the first edition of the *WHO Model list of essential medicines* in 1977 and the first edition of the *WHO Model list of essential medicines for children* in 2007, and extracted data from the entire set up to the 2021 updates (the 22nd and 8th editions, respectively).^19^^,^^20^ Two authors extracted data regarding the additions, deletions and rejections of medicines, including new formulations and new indications; and developed a pilot-tested Excel database (Microsoft Corp., Redmond, USA) for double-checking for inconsistency. We also retrieved data on orphan designation from publicly accessible databases of the FDA (1983–2020)^21^ and EU (2000–2020).^22^ We stopped here as the year 2020 was the deadline for submitting applications to the revised essential medicines lists in 2021. Additional information was collected from the Anatomical Therapeutic Chemical classification system;^23^ DrugBank^24^ for the type of product; MEDLINE to identify introduction of active pharmaceutical ingredients on markets; and MedsPal^25^ for patent status, a freely accessible database reporting patent data from roughly 130 countries worldwide. 

We considered all orphan drugs approved by the FDA and EMA from 1977 to 2021 with matching entries in all previous editions of the lists. All medicines we included had at least one orphan drug approval or indication from the FDA or EMA; some drugs might have received additional approvals for non-rare diseases. We considered a drug for inclusion if there was overlap of the following: (i) active pharmaceutical ingredient; (ii) pharmaceutical formulation; (iii) dosage; (iv) route of administration; and (v) therapeutic indication. Match pairing included medicines with a square box symbol which denotes therapeutic equivalence with other medicines in the same class.^26^ We reviewed each medicine and match, and discrepancies were discussed and overcome by involving a third author. New pharmaceutical versions of active pharmaceutical ingredients already listed in the essential list were analysed and discussed case by case (available in the online repository).^27^

### Data analysis

We used medicines approved by the FDA and EMA or included in the essential medicines lists as the unit of analysis. Medicines were stratified by therapeutic group based on the WHO anatomical therapeutic chemical classification and type of product. We evaluated the time-gap between the first publication of the active pharmaceutical ingredient in MEDLINE and its inclusion in the list as a measure of the dynamics that lead to the essential medicines list incorporation of emerging orphan drugs (Table 2). We also evaluated the patent status as a potential driver of price increases.^25^ We categorized drugs by date of inclusion in the essential medicines list. We also calculated median time to uptake in the list from first approval in either the FDA or EMA. 

**Table 2:** Orphan drugs approved by the United States FDA and the European Medicines Agency included in *WHO Model list of essential medicines* and *WHO Model list of essential medicines for* children, 2021

Anatomical therapeutic chemical group	FDA-approved orphan drugs			EMA-approved orphan drugs			No. of drugs in essential medicines lists	FDA- and EMA-approved orphan drugs in essential medicines lists
	No.	No. (%) in essential medicines lists		No.	No. (%) in essential medicines lists			
L: antineoplastic and immunomodulating agents	205	26 (12.7)		78	9 (11.5)		60	26 (43.3)
J: anti-infective for systemic use	53	13 (24.5)		11	3 (27.3)		130	14 (10.8)
P: antiparasitic products, insecticides and repellents	19	13 (68.4)		0	0 (0.0)		41	13 (31.7)
V: various	41	7 (17.1)		4	1 (25.0)		24	7 (29.2)
Other categories^a^	285	10 (3.5)		94	2 (2.1)		223	10 (4.5)
**Total**	**603**	**69 (11.4)**		**187**	**15 (8.0)**		**478**	**70 (14.6)**

EMA: European Medicines Agency; FDA: Food and Drug Administration; WHO: World Health Organization.

^a^ Includes N: nervous system; B: blood and blood-forming organs; C: cardiovascular system; H: systemic hormonal preparations, excluding sex hormones and insulins; S: sensory organs; R: respiratory system; M: musculo-skeletal system; A: alimentary tract and metabolism; D: dermatological; and G: genito-urinary system and sex hormones.

Diseases were clustered into communicable diseases and noncommunicable diseases. There is no universally agreed or WHO-endorsed definition of rare diseases whose prevalence cut-offs are arbitrary and valid within country or national limits. Epidemiological data for many rare diseases vary considerably across regions. Despite this, in 2005, WHO proposed describing different types of rare diseases in relation to essential medicines.^8^ We classified each pharmaceutical drug into one of four broad inclusion categories: (i) global (i.e. consistently rare across all world regions); (ii) regional (rare in one or more United Nations sub-regions but not in others);^28^ (iii) interim (rare in a specific time period); and (iv) special population (rare in a specific population, for instance children). We then compared orphan and non-orphan drug uptake in the essential medicines list over the same period to explore potential differences. We also explored why some applications were rejected for inclusion in the lists, and indexed them into five overlapping groups: disease; evidence; drug; use; and accessibility (further information is available in the online repository).^27^

## Results

### Trends and characteristics

**Fig. 1:**
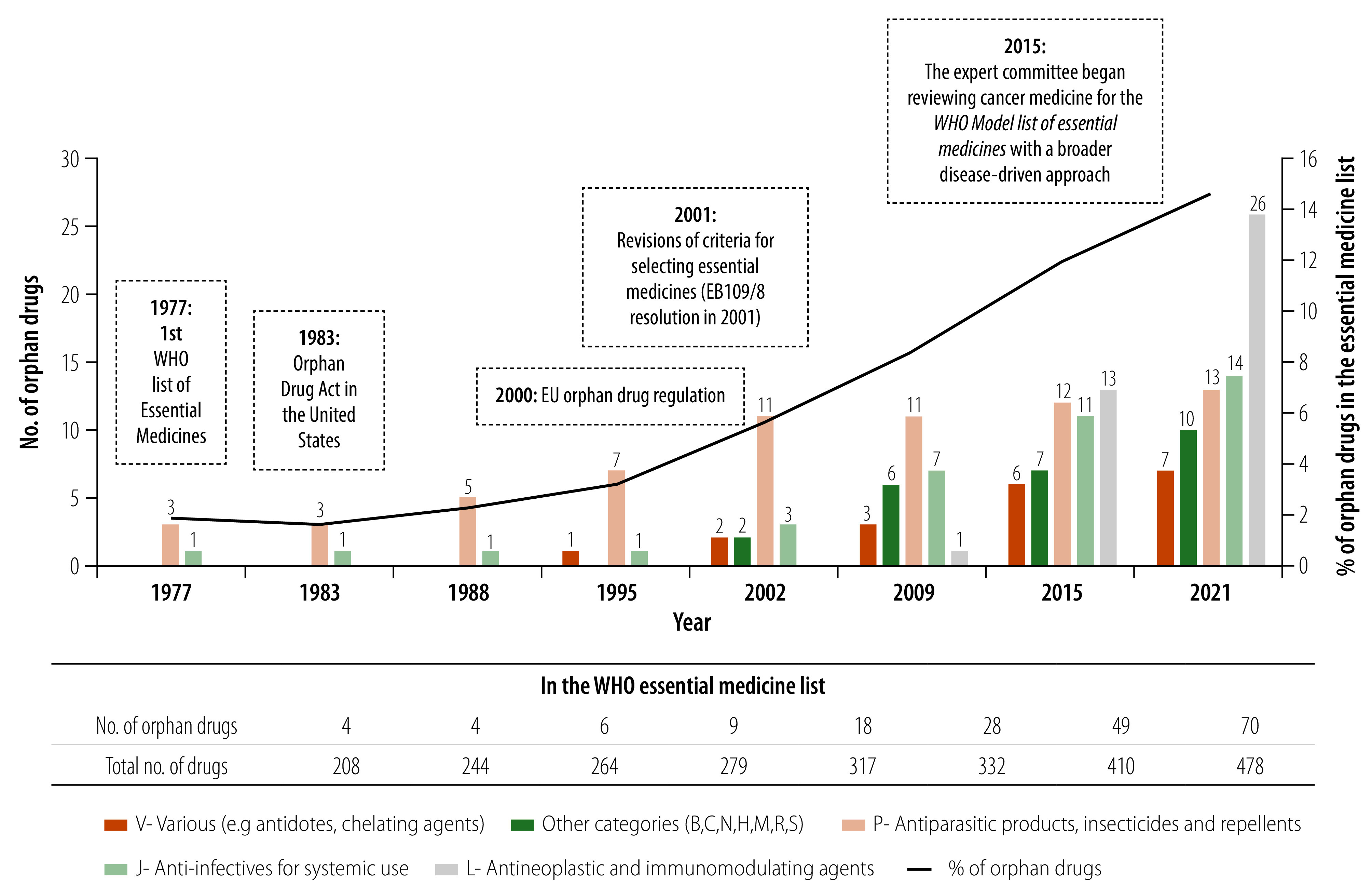
Types of orphan drugs included in WHO essential medicines lists, 1977–2021

EU: European Union; WHO: World Health Organization.

We noted an increase in the proportion of medicines with an orphan indication listed in the essential medicines lists that were approved by the FDA and/or EMA, from 1.9% (4/208) in 1977 to 14.6% (70/478) in 2021. While the number of orphan drugs, particularly for parasitic diseases, modestly increased during the 1980s and 1990s, since 2001, the number of orphan drugs listed on the essential medicines lists has more than tripled, covering a broader spectrum of therapeutic areas (Fig. 1). In 2021, among the 70 orphan drugs on the lists, 26 (37.1%) were categorized as antineoplastic and immunomodulating agents, 14 (20.0%) as anti-infectives, and 13 (18.6%) as antiparasitic products. 

This shift towards greater representation of orphan drugs on the essential medicines lists was also accompanied by an increase in the number of recently approved products with intellectual property protection. Specifically, in 2021, 19 out of the 70 orphan drugs (27.1%) had active main or secondary patents, compared to only 8.8% (36/408) for non-orphan drugs. Additionally, when we compared the time elapsed from the first indexed citation of the active substance of the orphan drug in the medical literature to its inclusion in the essential medicines lists, we found that the proportion with a time lag of less than 20 years was slightly higher for orphan drugs than for non-orphan drugs (41.4% versus 34.9%; Table 2).

### Differences between FDA and EMA

We observed that 98.6% (69/70) of orphan drugs included in the 2021 essential medicines list were approved by the FDA, whereas only 21.4% (15/70) were approved by the EMA. Fourteen drugs received approval from both the FDA and the EMA, while 55 were exclusively approved by the FDA, and only one was solely approved by the EMA. These disparities mirror the variations in the total number of orphan drugs approved in the USA and the European Union (EU), as well as the respective contributions of each jurisdiction to rare diseases. Specifically, 11.4% (69/603) of orphan drugs approved in the USA and 8.0% (15/187) in the EU were included in the essential medicines lists, with large differences across anatomical therapeutic chemical groups (Table 3).

**Table 3:** Characteristics of orphan drugs and non-orphan drugs listed in *WHO Model list of essential medicines* and *WHO Model list of essential medicines for children*, 2021

Characteristic	No. (%)		
	All essential medicines (*n* = 478)	Orphan drugs (*n* = 70)	Non-orphan drugs (*n* = 408)
***Essential medicines list for children***	351 (73.4)	57 (81.4)	294 (72.1)
***WHO Model list of essential medicines***			
Core	351 (73.4)	28 (40.0)	323 (79.2)
Complementary	127 (26.6)	42 (60.0)	85 (20.8)
**Product**			
Chemical	412 (86.2)	61 (87.1)	351 (86.0)
Biological	62 (13.0)	9 (12.9)	53 (13.0)
Device	4 (0.8)	0 (0.0)	4 (1.0)
**Patents**			
Active in most jurisdictions	27 (5.6)	11 (15.7)	16 (3.9)
Main expired but secondary active in some jurisdictions	28 (5.9)	8 (11.4)	20 (4.9)
Expired in most jurisdictions	405 (84.7)	51 (72.9)	354 (86.8)
Not Applicable	18 (3.8)	0 (0.0)	18 (4.4)
**MEDLINE to essential medicines list (years)^a^**			
≤ 20	171 (35.8)	29 (41.4)	142 (34.9)
21–50	225 (47.1)	24 (34.3)	201 (49.4)
> 51	72 (15.1)	17 (24.3)	55 (13.5)
Not Applicable	10 (2.1)	0 (0.0)	10 (2.5)
**Anatomical therapeutic chemical classification**			
J: Anti-infectives for systemic use	130 (27.2)	14 (20.0)	116 (28.4)
L: Antineoplastic and immunomodulating agents	60 (12.6)	26 (37.1)	34 (8.3)
P: Antiparasitic products. insecticides and repellents	41 (8.6)	13 (18.6)	28 (6.9)
V: Various (e.g. antidotes; chelating agents)	24 (5.0)	7 (10.0)	17 (4.2)
Other categories^b^	223 (46.7)	10 (14.3)	213 (52.2)

NA: not applicable.

^a^ Time between publications in MEDLINE and uptake in *WHO Model list of essential medicines*.

^b^ Includes N: nervous system; B: blood and blood-forming organs; C: cardiovascular system; H: systemic hormonal preparations, excluding sex hormones and insulins; S: sensory organs; R: respiratory system; M: musculo-skeletal system; A: alimentary tract and metabolism; D: dermatological; and G: genito-urinary system and sex hormones.

Our analysis shows that 12 orphan drugs which were initially included in the essential medicines list subsequently received orphan drug approval in the USA. Among these 12 orphan drugs, 11 were developed to address communicable diseases that are prevalent in regions with resource constraints. This subset consisted of seven drugs aimed at neglected tropical diseases and four intended for the treatment of malaria.

For the 58 medicines that were added to the essential medicines list following decisions by the FDA or EMA, the median time interval between their regulatory approval and inclusion in the list was approximately 13.5 years (range: 1–28 years).

### Targeted rare diseases

**Fig. 2:**
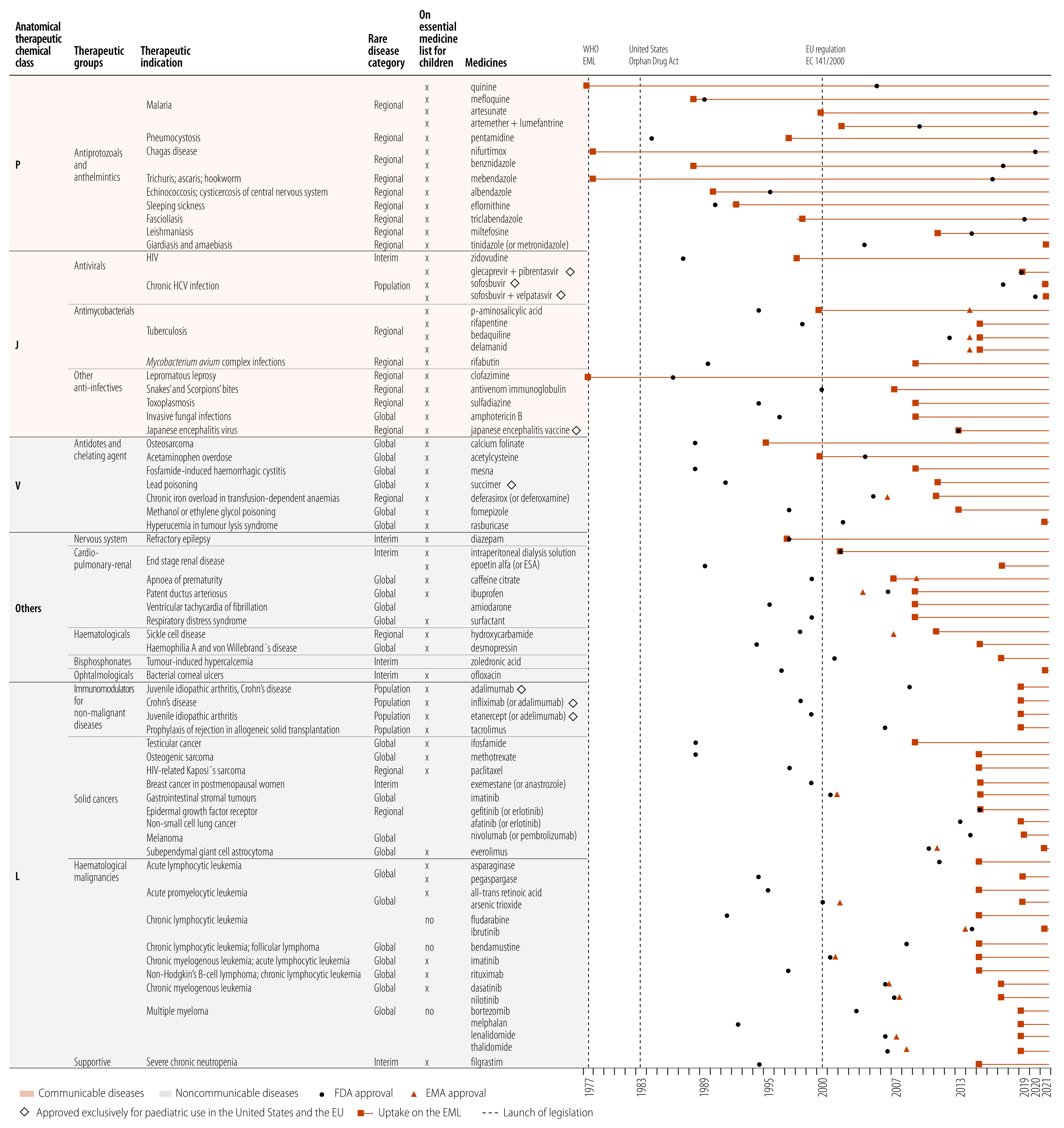
Orphan drug listings in the WHO essential medicines lists, 1977–2021

HCV: hepatitis C virus; HIV: human immunodeficiency virus; WHO: World Health Organization.

The FDA, EMA and WHO made decisions on 70 different orphan drugs recommended as essential medicines; 27 (38.6%) target communicable diseases including 10 drugs for neglected tropical diseases; four for malaria; four for tuberculosis; three for hepatitis C; and one for human immunodeficiency virus (HIV). The remaining 43 drugs (61.4%) target noncommunicable diseases, including 26 treating cancer (15 haematological malignancies, seven solid cancer, four supportive care), and 17 covering a wide spectrum of acute and chronic conditions. Furthermore, we noted that 31 (44.3%) of the orphan drugs target diseases that are rare worldwide, whereas 26 (37.1%) target diseases that are rare only in the USA and the EU but frequent in the rest of the world. For the rest, five (7.1%) target diseases that are overall relatively frequent but rare in special populations such as children, and eight (11.4%) target a condition (for example HIV) that is no longer rare (Fig. 2).

### Caveats and rejections

The inclusion of orphan drugs in the essential medicines lists has been accompanied by a range of precautionary recommendations aimed at ensuring the proper use of these orphan products. Notably, 42 (60.0%) orphan drugs are categorized within the complementary list, meaning that specialized diagnostic or monitoring facilities, specialist medical care, and/or specialist training are needed for their appropriate uptake into clinical practice. In contrast, this requirement is applicable to approximately 25% of non-orphan drugs included in the essential medicines list.

During the study period, 9.5% (25/262) of applications for orphan drugs were rejected by the WHO Expert Committee. On average, each application was rejected for two different reasons. Notably, concerns regarding price or cost were consistently accompanied by at least one concern regarding insufficient clinical evidence on net benefit or uncertainty related to the benefit. 

Interestingly, nine orphan drugs, previously rejected, were later resubmitted and added to the essential medicines lists. This inclusion occurred after a median duration of 2 years (interquartile range: 2–4 years), suggesting that any previous limitations were effectively addressed.

In cases where applications were rejected mainly due to price or costs, the subsequent inclusion of these drugs in the essential medicines lists was almost always influenced by the proposition of tiered pricing for low- and middle-income countries. Additionally, the availability of generic versions of these drugs plays a substantial role in their eventual inclusion into the essential medicines lists (online repository).^27^

## Discussion

Orphan drugs are a historical prerogative for high-income countries because of their niche targets, complexity of clinical use and high cost. We observed an increase in the uptake of orphan drugs on the essential medicines lists over the study period. Before 2000, orphan drugs were rarely recommended by the essential medicines lists, and those listed mostly targeted communicable diseases such as malaria and other neglected tropical diseases, that are rare in the United States and the EU but more frequent in low- and middle-income countries. The steady increase in the number of orphan drugs over the last two decades includes drugs for noncommunicable diseases such as cancer. This increase is consistent with global health challenges and priorities in almost all international agendas.^29^ Between 1977 and 2021, global investments in research for rare diseases led to a rapid increase in the number of orphan drugs licensed in both the USA and the EU. Among them, a relevant proportion fulfilled the criteria as essential medicines.

The United States and EU orphan drug systems have conceptual and temporally distinct origins which have influenced the number of orphan drugs approved within each set of regulations. Medicines approved as orphan drugs by the FDA might be approved without orphan designation in the EU, where the criteria for granting such designations is more stringent.^30^ Over the last decade, both the FDA and EMA introduced specialized programmes to expedite the reviewing and approval of medicines, allowing them to accept flexible and/or less comprehensive evidence compared to normal requirements.^31^ The rapid assessment of the FDA and EMA might contrast with the preconditions to be listed as essential medicines, the maturity and certainty of evidence for substantial benefits and safety, and contribute to a long time lag between orphan drug designation and essential medicines listing. The conceptual and pragmatic differences in evaluating medicines legitimize why certain medicines have not been recommended by all three entities. Our study found that the most frequent reason for rejection by the essential medicines lists of drugs approved by the FDA or the EMA was insufficient clinical evidence on net benefit or uncertainty related to the benefit.

The uptake in the essential medicines lists does not guarantee the product's presence on every national reimbursement list worldwide. The essential medicines lists serve as model lists. A decision to include the product will be made on a national level, based on epidemiology, health priorities and resources, sometimes in multicountry arrangements.^32^^,^^33^ Noteworthy is that 60% of study drugs included in the essential medicines lists are listed in the complementary list, thus requiring more specialized expertise and adequate facilities for their appropriate use. This issue poses serious challenges in countries where skilled personnel and equipped centres with adequate diagnostic and technical infrastructure are inadequate. In our study we only considered orphan designation based on the United States and European regulatory authorities. Other world regions were not represented, and no conclusions can be drawn about orphan designations in these regions and the essential medicines list status.

WHO takes international clinical guidelines and health systems capacity of low- and middle-income countries into consideration when deciding about the inclusion of medicines on the essential medicines lists. Limitations related to the feasibility of implementing the treatment was the most common reason for rejecting requests to add orphan drugs to the list. For example, for target therapy (e.g. tyrosine-kinase inhibitors for lung cancer), appropriate prescriptions require the detection of specific mutations by means of molecular diagnostic tools, whose availability is jeopardized in low- and middle-income countries' laboratories.^34^ An early application for tyrosine-kinase inhibitors was rejected by the essential medicines list. Once the availability of molecular diagnostics was sufficiently widespread, this facilitated the inclusion of tyrosine-kinase inhibitors. Lastly, the availability of generic or biosimilar products played a similar role, as this allows low- and middle-income countries to procure the generic version for the study drug at a more affordable price for their health system and population. The cost of the proposed treatment, whether this referred to high prices and/or costs, or lack of price and/or cost information was another frequent reason for rejection.

Diseases patterns and therapeutics approaches have considerably changed since the inception of the essential medicines list. Cancer is an illustrative example. For a long time, the disease was considered as an exclusive public health priority in high-income countries. Nowadays cancer ranks as a leading cause of death in most countries.^29^ As new effective medicines for cancer became available, WHO intensified their reviewing of cancer medicines. Today around one-third of the orphan drugs on the essential medicines list targets oncological malignancies.^35^^,^^36^ Zidovudine, the first pharmaceutical agent licensed to target HIV in the 1990s, was marketed as an orphan drug by the FDA, as at that time the prevalence of people living with HIV in the USA was well below the threshold set by the Orphan Drug Act.^37^ Over the past decades, migrations and global population dynamics have changed the geographical distribution of diseases. Neglected tropical diseases, tuberculosis, malaria and haemoglobinopathies – conditions yearly affecting millions of people worldwide – might be eligible for orphan incentives in the USA and the EU when diseases prevalence falls below the threshold provided by orphan legislations.^36^

The 2001 change of the inclusion criteria made it possible for the lists of essential medicines to recommend an increasing number of medicines for rare diseases that came onto market under the stimulus of orphan legislations in the US and the EU. While this change is promising for rare disease patients, it poses significant challenges in implementing a comprehensive essential medicines list at the national level, including diagnostic capacity and expertise. These broadened inclusion criteria might discourage some policy-makers from using the essential medicines lists as a guideline for the treatment-related health-care needs of the community or population.^38^^,^^39^ Novel and ground-breaking innovations such as treatments to correct very rare metabolic diseases in paediatric patients, and gene or cell-based therapies may further exacerbate the tension between highly effective orphan drugs and related financial burdens.^40^^,^^41^ In a world where a global definition for rare diseases remains elusive, the role of the essential medicines list is central to orient decisions for equitable access to medicines, especially for rare disease patients. Future studies should examine the impact of orphan drug essential medicines list inclusion on national adoption and availability.
